# Abdominal aortic calcification score as a predictor of clinical outcome in peritoneal dialysis patients: a prospective cohort study

**DOI:** 10.1186/s12882-020-01822-9

**Published:** 2020-04-30

**Authors:** Dahua Ma, Hao Yan, Xiaoxiao Yang, Zanzhe Yu, Zhaohui Ni, Wei Fang

**Affiliations:** 1 Department of Nephrology, Renji Hospital, School of Medicine, Shanghai Jiao Tong University, No. 160, Pujian Road, Pudong District, Shanghai, 200127 People’s Republic of China; 2 Shanghai Center for Peritoneal Dialysis Research, Shanghai, China

**Keywords:** Abdominal aortic calcification score, Major adverse cardiovascular and cerebrovascular events, All-cause mortality, Peritoneal dialysis

## Abstract

**Background:**

Abdominal aortic calcification assessed by X-ray is recommended to evaluate vascular calcification in dialysis patients. It has been shown that abdominal aortic calcification score (AACS) is a predictor of adverse outcomes in hemodialysis patients, but evidence regarding its prognostic value in peritoneal dialysis (PD) patients is still insufficient. We aimed to examine the predictive role of AACS for major adverse cardiac and cerebrovascular events (MACCE) and mortality in PD patients.

**Methods:**

Eligible patients undergoing PD between July 2011 and July 2014 were recruited. AACS was quantified using lateral lumbar radiography at recruitment. Patients were prospectively followed up until death, PD cessation, or to the end of the study (August 31, 2018). Both subdistribution hazards and cause-specific hazards models were used to evaluate the association between AACS and MACCE as well as mortality.

**Results:**

292 patients were enrolled, including 160 males (54.8%) with mean age 57.1 ± 15.2 years and median PD duration 28.4 (IQR 12.0, 57.8) months. Among them, 75 (25.7%) patients were comorbid with diabetes, and 94 (32.2%) patients had cardiovascular disease (CVD). The average AACS was 2.0 (0.0, 6.0). Patients were categorized on the tertiles of AACS (Low AACS group, AACS = 0, *n* = 125; Medium AACS group, AACS 1–4, *n* = 72; and High AACS group, AACS> 4, *n* = 95). AACS was associated with age (OR = 1.081, *P* < 0.001), PD duration (OR = 1.012, *P* = 0.003), CVD (OR = 1.919, *P* = 0.020) and diabetes (OR = 2.554, *P* = 0.002). During the follow-up period of 43.6 (24.6, 50.7) months, there were 65 MACCEs and 84 deaths. Significantly higher cumulative incidences of all-cause mortality (Log-rank = 35.992, P<0.001; Gray = 38.662, *P* < 0.001) and MACCE (Log-rank = 26.146, P<0.001; Gray = 27.810, P < 0.001) were observed in the upper AACS tertile. AACS was an independent predictor of all-cause mortality (HR = 2.438, 95% CI 1.246–4.772, *P* = 0.009; SHR = 2.323, 95%CI 1.229–4.389, P = 0.009) and MACCE (HR = 3.455, 95% CI 1.734–6.884, *P* < 0.001; SHR = 3.063, 95%CI 1.460–6.430, *P* = 0.003) in this study.

**Conclusions:**

AACS was associated with age, PD duration, CVD and diabetes in PD patients. AACS could predict MACCE and all-cause mortality in this population. It thus might be a safe and feasible method to identify PD patients with adverse outcomes.

## Background

Arterial calcification is associated with great risks of cardiovascular disease (CVD) among patients with different chronic kidney disease (CKD) stages, which translates into excess cardiovascular mortality in this population [1, 2]. It has been well established that vascular calcification is more prevalent in CKD patients, especially those on dialysis, than that in the age-matched general population [3, 4]. Several imaging techniques have been utilized in the evaluation of vascular calcification, such as ultrasonography, computed tomography (CT), mammograms and plain radiographs [5–7]. In dialysis patients, abdominal aortic calcification score (AACS) based on lateral lumbar X-Ray has been recommended to evaluate the extent of vascular calcification [8]. The AACS scale developed by Kauppila et al could independently predict all-cause mortality and nonfatal CVD events in hemodialysis (HD) pateints [9, 10], whereas there was limited clinical evidence to verify its prognostic value in patients undergoing peritoneal dialysis (PD).

Therefore, this study was conducted to explore the association of AACS with major adverse cardiovascular and cerebrovascular events (MACCE) and all-cause mortality in prevalent Chinese PD patients in a large university teaching hospital.

## Methods

### Patients and study design

This was a single-center, prospective, cohort study. Patients aged at least 18 years who had been on PD for more than 3 months between July 2011 and July 2014 were recruited. Exclusion criteria were as follows: (1) with active infections, malignancy or pregnancy at the time of recruitment; (2) hybrid HD and PD; (3) refusal to provide consent. Eligible PD patients were enrolled and AACS was quantified in all patients using lateral lumbar radiography at recruitment. The patients were followed up until death, switch to hemodialysis, kidney transplantation, dialysis-independent renal recovery, transfer to other centers, or until August 31, 2018. All the patients were dialyzed with lactate-buffered glucose-based PD solutions (Dianeal®, Baxter, China).

### Clinical data collection

At the time of enrollment, participants’ demographic characteristics, underlying end-stage renal disease (ESRD) cause, PD duration, comorbidity status, laboratory parameters, PD regimen, and concomitant medications were recorded. Diabetes mellitus was defined either as a comorbid condition or as the aetiology of ESRD. Hypertension was determined as blood pressure consistently higher than 140/90 mmHg or have been on the treatment of hypertension medication. Pre-existing cardiovascular disease was defined as the history of any undermentioned condition: acute coronary syndrome, heart failure, cerebral infarction or hemorrhage, coronary artery atherosclerosis confirmed by percutaneous coronary intervention (PCI) or coronary artery bypass grafting (CABG) therapy.

Laboratory parameters including hemoglobin, high-sensitivity C reaction protein (hs-CRP), serum albumin, lipid profiles, calcium, phosphate, intact parathyroid hormone (iPTH) and alkaline phosphatase (AKP) were evaluated. Adequacy of dialysis was estimated by measuring total weekly urea clearance (Kt/V) by standard methods [11]. Residual renal function was calculated as an average of 24-h urinary urea and creatinine clearance [12]. At the same time, baseline lateral lumbar X-Ray film was performed, and AACS was scored for each individual by two experienced radiologists who were blinded to the patients’ clinical data using a specific scale previously described [6]. Patients were then divided into 3 groups according to AACS tertiles.

### Outcomes

The primary outcome was all-cause mortality. The secondary outcome was the first MACCE during the follow-up. MACCE was defined as follows: fatal or non-fatal acute coronary syndrome (ACS), angina requiring coronary revascularization either by PCI or CABG, acute heart failure requiring hospitalization, sudden death, transient or permanent neurologic deficit with image evidence of cerebral ischemic/hemorrhagic lesion on computed tomography or magnetic resonance.

### Statistical analysis

Continuous variables were reported as mean ± standard deviation (SD) for normal distribution or median (interquartile range [IQR]) for skewed distribution, and categorical variables as frequencies (percentages). Continuous variables were compared among groups by One-way ANOVA analysis or Kruskal-Wallis test, and categorical variables by Chi-square (χ2) test. Associated factors of abdominal aortic calcification were examined using a stepwise multivariable logistic model, and variables with a *p* value < 0.1 were included in the model except those with multicollinearity.

Considering the presence of competing events in this study, we additionally performed survival analysis by reporting subdistribution hazards attached to cumulative incidence [13]. For univariate analysis, both Kaplan-Meier and cumulative incidence competing risk (CICR) methods were used to estimate the probabilities of mortality and MACCE, and differences among groups were compared by the Log-rank test and Gray test respectively. For Multivariate analysis, cause-specific hazards and subdistribution hazards models were used to explore relative risks of all-cause mortality and MACCE for different variables. When the event of interest was all-cause mortality, the competing events included switch to HD, receiving kidney transplant and transfer to other centers. When the event of interest was MACCE occurrence, the competing events included switch to HD, receiving kidney transplant, transfer to other centers and death unrelated to MACCE.

Data analysis was performed using SPSS for windows version 25 (IBM Corporation, Armonk, NY) and R for windows vision 3.6.1. All probabilities were two-tailed, and *P*-value < 0.05 was considered statistically significant.

## Results

### Patient characteristics and associated factors of AACS

The cohort consisted of 292 PD patients, among whom 160 (54.8%) were males. The mean age was 57.1 ± 15.2 years, and the median PD duration was 28.4 (12.0, 57.8) months. Among them, 75 (25.7%) patients had diabetes and 94 (32.2%) had pre-existing cardiovascular disease. The demographic characteristics, ESRD etiologies, comorbidity status, and baseline laboratory parameters were summarized in Table 1. A history of cardiovascular or cerebrovascular disease before enrollment was identified in 94 (32.2%) patients, including ischemic stroke (*n* = 30), hemorrhagic stroke (*n* = 7), subarachnoid hemorrhage (*n* = 1), myocardial infarction (*n* = 7), congestive heart failure (*n* = 49), ischemic heart disease requiring CABG (*n* = 2), and angina (*n* = 19). Some patients(n = 4) had had multiple CVD strikes.

**Table 1:** Clinical characteristics of patients across tertiles of AACS

	Total (*n* = 292)	Low AACS group (*n* = 125)	Medium AACS group (*n* = 72)	High AACS group (*n* = 95)	*P* Value
Age (year)	57.1 ± 15.2	48.5 ± 14.9	59.8 ± 12.1	66.4 ± 10.8	< 0.001
Male gender, n (%)	160 (54.8)	67 (46.4)	37 (51.4)	56 (58.9)	0.586
BMI (kg/m^2^)	22.4 (20.3, 25.0)	21.6 (19.7, 24.5)	22.5 (21.0, 25.5)	23.1 (21.4, 25.5)	0.013
Dialysis Duration (month)	28.4 (12.0, 57.8)	21.7 (7.6, 52.7)	29.2 (13.6, 56.9)	41.7 (19.0, 71.0)	0.004
CKD etiology, n (%)					0.105
Glomerulonephritis	103(35.3%)	51(40.8%)	24(33.3%)	28(29.5%)	
Polycystic kidney	9(3.1%)	5(4.0%)	3(4.2%)	1(1.1%)	
Diabetic nephropathy	43 (14.7%)	7 (5.6)	15 (20.8)	21 (22.1)	
Hypertension	12(4.1%)	5(4.0%)	3(4.2%)	4(4.2%)	
Others	10(3.4%)	5(4.0%)	1(1.4%)	4(4.2%)	
Unknown	115(39.4%)	52(41.6%)	26(36.1%)	37(38.9%)	
Hypertension, n (%)	270 (92.5%)	110 (88.0%)	69 (95.8%)	91 (95.8%)	0.044
Diabetes, n (%)	75 (25.7%)	13 (10.4%)	24 (33.3%)	38 (50.7%)	< 0.001
CVD history, n (%)	94 (32.2%)	20 (16.0%)	28 (38.9%)	46 (48.9%)	< 0.001
Total Kt/V	1.82 (1.61, 2.11)	1.84 (1.63, 2.18)	1.82 (1.65, 2.05)	1.81 (1.58, 2.07)	0.292
RRF (ml/min)	0.9 (0.0, 2.9)	1.0 (0.0, 3.7)	0.7 (0.0, 2.7)	0.8 (0.0, 2.0)	0.204
Urine volume (ml/d)	300 (0, 800)	375 (0, 988)	300 (0, 725)	280 (0, 600)	0.167
Albumin (g/L)	37.4 (34.6, 40.5)	38.6 (35.4, 41.5)	37.8 (34.3, 40.4)	37.2 (33.2, 39.7)	0.031
hs-CRP (mg/L)	2.3 (0.7, 5.8)	1.4 (0.6, 4.5)	2.9 (1.0, 6.5)	3.4 (1.2, 7.6)	0.002
Corrected calcium (mmol/L)	2.35 ± 0.20	2.32 ± 0.18	2.37 ± 0.21	2.37 ± 0.21	0.137
Phosphorus (mmol/L)	1.54 (1.26, 1.84)	1.52 (1.28, 1.87)	1.47 (1.21, 1.77)	1.60 (1.25, 1.85)	0.789
iPTH (pg/ml)	274 (126, 568)	323 (131, 595)	300 (99, 527)	242 (130, 531)	0.659
AKP (U/L)	85.0 (65.0, 113.0)	86.5 (65.0, 109.8)	80.0 (64.0, 104.8)	89.0 (66.0, 126.0)	0.221
TG (mmol/L)	1.82 (1.23, 2.79)	1.65 (1.14, 2.30)	1.93 (1.24, 2.41)	2.21 (1.36, 3.26)	0.007
TC (mmol/L)	5.08 ± 1.19	4.99 ± 1.11	5.02 ± 1.09	5.22 ± 1.35	0.346
LDL (mmol/L)	2.86 (2.18, 3.51)	2.68 (2.18, 3.62)	2.98 (2.06, 3.58)	2.96 (2.21,3.38)	0.963
HDL (mmol/L)	1.11 (0.90, 1.46)	1.21 (0.95, 1.51)	1.11 (0.90, 1.40)	1.00 (0.86, 1.39)	0.002
Use of calcium-based phosphate binder, n (%)	210 (72.2%)	94 (75.2%)	46 (64.8%)	70 (73.7%)	0.272
Use of Low-calcium dialysate, n (%)	114 (39.2%)	48(38.4%)	24(33.8%)	42(44.2%)	0.386

Abbreviations: *AACS* abdominal aortic calcification score, *BMI* body mass index, *CVD* cardiovascular disease, *RRF* residual renal function, hs-CRP: high-sensitivity C reaction protein, *TC* total cholesterol, *TG* total triglyceride, *HDL* high-density lipoprotein, *LDL* low- density lipoprotein, Corrected calcium: serum total calcium (corrected by albumin), *iPTH* intact-parathyroid hormone, *AKP* alkaline phosphatase. Calcium-based phosphate binder refers to Calcium Carbonate and Calcium Acetate

Abdominal artery calcification was observed in 167 (57.2%) participants according to the results of lateral lumbar X-ray film. The median AACS of the entire cohort was 2.0 (0.0, 6.0). Patients were divided into 3 groups according to the tertiles of AACS: Low AACS group, AACS = 0, *n* = 125; Medium AACS group, AACS 1–4, *n* = 72; and High AACS group, AACS> 4, *n* = 95.

Participants with high AACS were more likely to be older, with higher BMI and a longer PD duration, more prevalent in hypertension, diabetes, and CVD, with lower serum albumin and HDL, but greater hs-CRP and TG (Table 1). Multivariate linear regression revealed that older age (OR 1.081, 95% CI 1.056–1.107, *P* < 0.001), longer PD duration (OR 1.012, 95% CI 1.004–1.019, *P* = 0.003), presence of diabetes (OR 2.554, 95% CI 1.415–4.609, *P* = 0.002) and previous CVD (OR 1.919, 95% CI 1.108–3.325, *P* = 0.020) were associated with high AACS (Table 2).

**Table 2:** Potential risk factors of greater AACS in the study participants

	β	OR (95% CI)	*P* Value
Age (year)	0.078	1.081 (1.057,1.106)	< 0.001
Male gender	0.141	1.152(0.699,1.897)	0.579
BMI (kg/m^2^)	−0.011	0.989 (0.927,1.055)	0.730
Dialysis Duration (month)	0.012	1.012(1.004,1.019)	0.003
Hypertension	0.385	1.469 (0.527,4.098)	0.462
Diabetes	0.938	2.554 (1.415,4.609)	0.002
CVD history	0.652	1.919 (1.108,3.325)	0.020
Albumin (g/L)	0.027	1.027 (0.968,1.089)	0.376
hs-CRP (mg/L)	0.015	1.015 (0.987,1.043)	0.295
RRF (ml/min)	−0.104	0.901 (0.796,1.021)	0.101

Abbreviations: *BMI* body mass index, *CVD* cardiovascular disease, *hs-CRP* high-sensitivity C reaction protein, *RRF* residual renal function, *OR* odds ratio

### Association between AACS and all-cause mortality

After the median follow-up of 43.6 (24.6, 50.7) months, 84 (28.8%) patients had died, 42 (14.4%) had been switched to hemodialysis, 22 (7.5%) had received kidney transplantation, and 11 (3.8%) had transferred to other centers.

A total of 50 patients (59.52%) died because of a lethal MACCE strike, which was the leading cause of death, including 28 cases of sudden death, 7 of acute myocardial infarction, 7 of cerebral hemorrhage, 6 of cerebral infarction, and 2 of decompensated heart failure. Infection was the second mortality cause (*n* = 23), including peritonitis in 9 patients. The other deceased died from gastrointestinal bleeding (*n* = 5), malignancy (*n* = 1), or unknown causes (n = 5). AACS was greater in the dead than that in the survivors [5.0 (1.0, 9.0) vs. 0.0 (0.0, 4.0), *P* < 0.001].

**Fig. 1:**
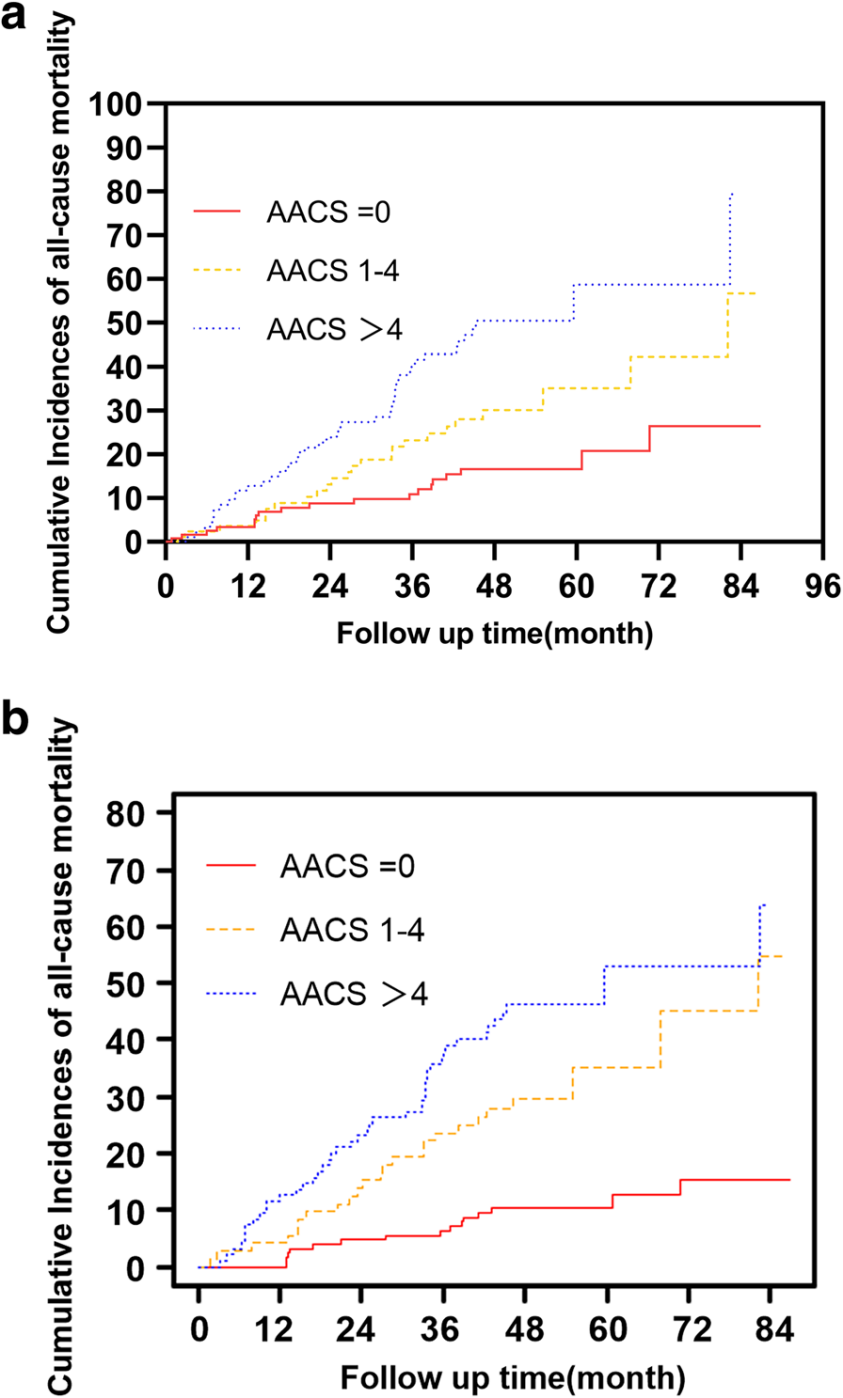
Cumulative incidences of all-cause mortality estimated by **a** Kaplan-Meier (Log-rank = 35.992, *P* < 0.001) and **b** cumulative incidence competing risk (CICR) methods (Gray = 38.662, P < 0.001) of patients stratified by AACS tertiles

The estimated cumulative mortality incidences were significantly lower in patients of Low AACS group than in their counterparts of Medium and High AACS group (Log-rank = 35.992, *P* < 0.001, Fig.1a; Gray = 38.662, *P* < 0.001, Fig.1b). The multivariate Cox regression model showed that the baseline AACS independently predicted all-cause mortality (Medium AACS group vs. Low AACS group: HR 2.028, 95% CI 1.014–4.057, *P* = 0.046; High AACS group vs. Low AACS group: HR 2.438, 95% CI 1.246–4.772, *P* = 0.009) after adjusting for age, gender, BMI, hypertension, diabetes, previous CVD, PD duration, total Kt/V, serum albumin, TG, and use of calcium-based phosphate binders (Table 3). In the presence of competing events including switch to HD, receiving kidney transplant and transfer to other centers, severe abdominal artery calcification remained independently predictable of all-cause mortality (Medium AACS group vs. Low AACS group: SHR 1.772, 95% CI 0.899–3.496, *P* = 0.099; High AACS group vs. Low AACS group: SHR 2.323, 95%CI 1.229–4.389, *P* = 0.009; Table 4).

**Table 3:** Cause-specific hazards model of different variables on all-cause mortality

Variable	Univariate Analysis		Multivariate Analysis	
	HR (95% CI)	*P* Value	HR (95% CI)	*P* Value
Age (year)	1.054(1.036,1.037)	< 0.001	1.035(1.012,1.058)	0.002
Male Gender	0.920(0.605,1.398)	0.695	–	–
Diabetes	2.500(1.631,3.831)	< 0.001	–	–
Dialysis Duration (month)	0.996(0.990,1.002)	0.226	–	–
Hypertension	4.657(1.140,19.025)	0.032	–	–
CVD history	3.499(2.287,5.353)	< 0.001	2.146(1.351,3.407)	0.001
BMI (kg/m^2^)	1.050(0.997,1.107)	0.067	–	–
Albumin(g/L)	0.905(0.866,0.946)	< 0.001	0.932(0.882,0.984)	0.012
Kt/V	0.482(0.263,0.884)	0.018	–	–
TG (mmol/L)	1.115 (1.011,1.230)	0.030	1.131(1.014,1.261)	0.027
Calcium-based phosphate binders	0.676 (0.437,1.043)	0.077	–	–
Medium vs. Low AACS group	3.374(1.764,6.452)	< 0.001	2.028(1.014,4.057)	0.046
High vs. Low AACS group	5.200(2.886,9.369)	< 0.001	2.438(1.246,4.772)	0.009

Abbreviations: *CVD* cardiovascular disease, *BMI* body mass index, *TG* total triglyceride, *HR* hazard ratio. Calcium-based phosphate binder refers to Calcium Carbonate and Calcium Acetate

**Table 4:** Subdistribution hazards model of different variables on all-cause mortality

Variable	Univariate Analysis		Multivariate Analysis	
	SHR (95% CI)	*P* Value	SHR (95% CI)	*P* Value
Age (year)	1.060(1.040, 1.080)	< 0.001	1.040(1.012, 1.068)	0.004
Male Gender	0.927(0.605, 1.420)	0.730	–	–
Diabetes	2.650(1.720, 4.080)	< 0.001	–	–
Dialysis Duration (month)	0.997(0.992, 1.000)	0.350	–	–
Hypertension	8.120(1.10, 60.200)	0.040	–	–
CVD history	3.380(2.200, 5.190)	< 0.001	–	–
BMI (kg/m2)	1.040(0.987, 1.100)	0.130	–	–
Albumin (g/L)	0.905(0.856, 0.947)	< 0.001	0.946(0.896, 0.999)	0.046
hs-CRP (mg/L)	1.010(1.000, 1.020)	0.003	–	–
Kt/V	0.519(0.265, 1.020)	0.056	–	–
TG (mmol/L)	1.090(0.999, 1.200)	0.052	–	–
Calcium-based phosphate binders	0.600(0.387, 0.931)	0.023	–	–
Medium vs. Low AACS group	3.350(1.770,6.350)	< 0.001	1.772(0.899, 3.496)	0.099
High vs. Low AACS group	5.460(3.050, 9.780)	< 0.001	2.323(1.229, 4.389)	0.009

Abbreviations: *CVD* cardiovascular disease, *BMI* body mass index, *hs-CRP* high-sensitivity C reaction protein, *TG* total triglyceride, *SHR* subdistribution hazard ratio. Calcium-based phosphate binder refers to Calcium Carbonate and Calcium Acetate

### The association between AACS and MACCE

MACCE occurred in 65 (22.3%) patients during the follow-up, including ACS (*n* = 14), acute left ventricular failure (*n* = 3), cerebral infarction (*n* = 14), cerebral hemorrhage (*n* = 10), and sudden cardiac deaths (*n* = 24). Patients who developed MACCE showed higher AACS than the others did [4.5 (1.0, 8.0) vs. 1.0 (0.0, 5.0), *p* < 0.001].

**Fig. 2:**
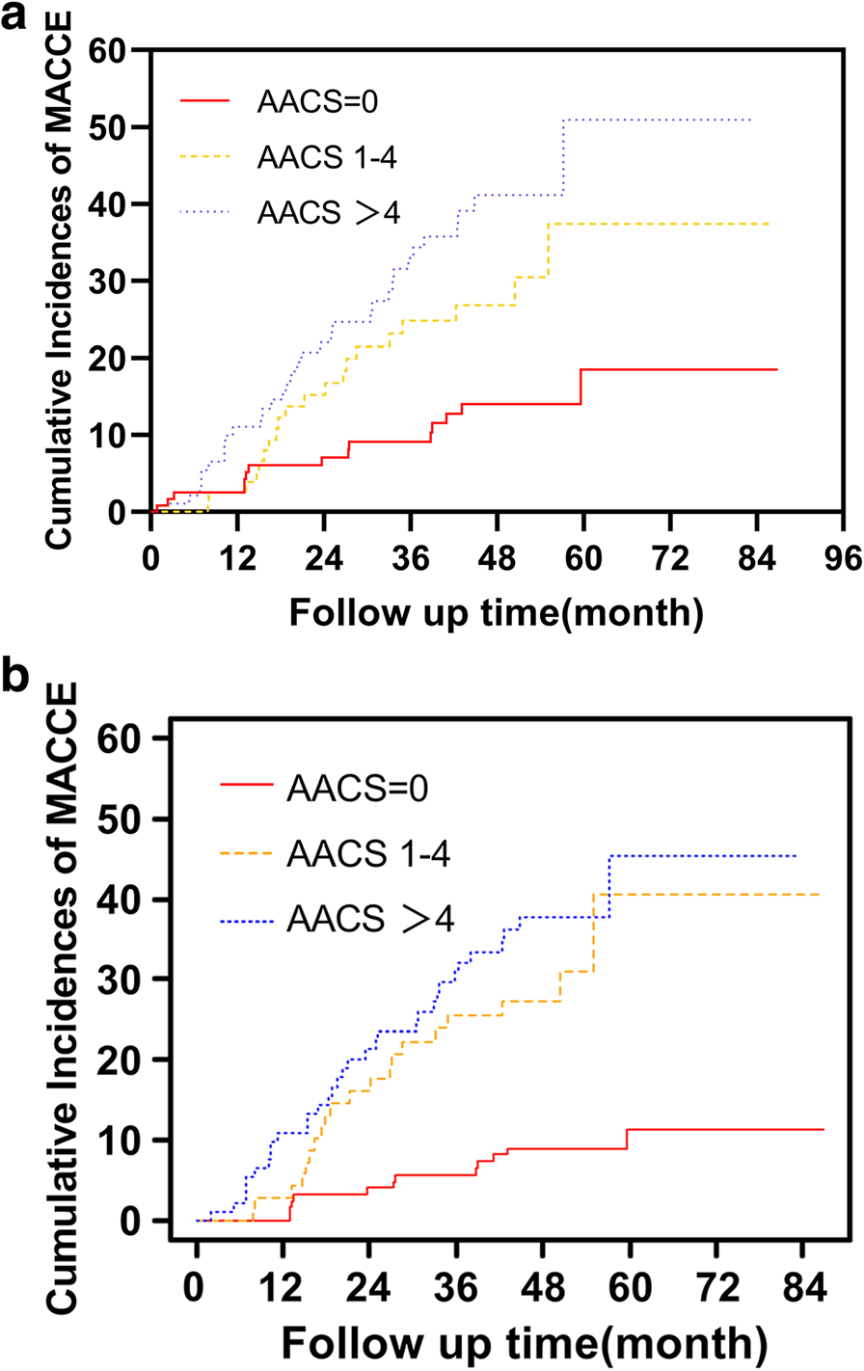
Cumulative incidences of MACCE estimated by **a** Kaplan-Meier (Log-rank = 26.146, P < 0.001) and **b** cumulative incidence competing risk (CICR) methods (Gray = 27.810, P < 0.001) of patients stratified by AACS tertiles

Patients of the Low AACS group had significantly lower cumulative incidences of MACCE (Log-rank = 26.146, *P* < 0.001; Fig.2a; Gray = 27.810, *P* < 0.001, Fig.2b). After adjusting for age, gender, BMI, hypertension, diabetes, previous CVD, PD duration, total Kt/V, serum albumin, and LDL-c, multivariate Cox regression analysis presented the baseline AACS as an independent predictor of MACCE (Medium AACS group vs. Low AACS group: HR 2.976, 95% CI 1.420–6.238, *P* = 0.004; High AACS group vs. Low AACS group: HR 3.455, 95% CI 1.734–6.881, *P* < 0.001; Table 5). Further adjusted by competing events including switch to HD, receiving kidney transplant, transfer to other centers and death unrelated to MACCE, AACS remained as an independent predictor of MACCE (Medium AACS group vs. Low AACS group: SHR 2.823, 95% CI 1.333–5.970, *P* = 0.007; High AACS group vs. Low AACS group: SHR 3.063, 95%CI 1.460–6.430, *P* = 0.003; Table 6).

**Table 5:** Cause-specific hazards model of different variables on MACCE

	Univariate Analysis		Multivariate Analysis	
	HR (95% CI)	*P* value	HR (95% CI)	*P* Value
Age (year)	1.041 (1.021, 1.061)	< 0.001	1.011(0.986,1.035)	0.396
Male Gender	1.183 (0.722, 1.938)	0.504	–	–
Dialysis Duration (month)	0.993 (0.985, 1.000)	0.067	–	–
Diabetes	2.328 (1.419, 3.819)	0.001	–	–
Hypertension	2.176 (0.777, 12.991)	0.108	–	–
CVD history	2.677 (1.647, 4.352)	< 0.001	2.221(1.324, 3.726)	0.003
BMI (kg/m^2^)	1.065 (1.004, 1.129)	0.035	–	–
Albumin(g/L)	0.932 (0.885, 0.981)	0.007	–	–
Total Kt/V	0.528 (0.259, 1.074)	0.078	–	–
LDL (mmol/L)	1.276 (1.083, 1.504)	0.004	1.269(1.056,1.525)	0.011
Medium vs. Low AACS group	4.830 (2.493, 9.436)	< 0.001	2.976(1.420,6.238)	0.004
High vs. Low AACS group	3.799 (1.845, 7.824)	< 0.001	3.455(1.734,6.884)	0.000

Abbreviations: *CVD* cardiovascular disease, *BMI* body mass index, *LDL* low- density lipoprotein, *HR* hazard ratio

**Table 6:** Subdistribution hazards model of different variables on MACCE

	Univariate Analysis		Multivariate Analysis	
	SHR (95% CI)	*P* Value	SHR (95% CI)	*P* Value
Age (year)	1.040 (1.020, 1.060)	< 0.001	–	–
Male Gender	1.140 (0.698, 1.870)	0.600	–	–
Dialysis Duration (month)	0.994(0.987, 1.000)	0.070	–	–
Diabetes	2.390 (1.450, 3.910)	< 0.001	–	–
Hypertension	3.050 (0.763, 12.200)	0.110	–	–
CVD history	2.840 (1.750, 4.630)	< 0.001	1.790 (1.024, 3.130)	0.041
BMI (kg/m^2^)	1.060 (1.010, 1.130)	0.026	–	–
Albumin(g/L)	0.930 (0.884, 0.979)	0.005	–	–
hs-CRP (mg/L)	1.010 (1.000, 1.020)	0.022	–	–
LDL (mmol/L)	1.320 (1.070, 1.640)	0.011	1.330 (1.080, 1.640)	0.007
Medium vs. Low AACS group	3.710 (1.820, 7.570)	< 0.001	2.823(1.333, 5.970)	0.007
High vs. Low AACS group	5.000 (2.590, 9.640)	< 0.001	3.063(1.460, 6.430)	0.003

Abbreviations: *CVD* cardiovascular disease, *BMI* body mass index, *hs-CRP* high-sensitivity C reaction protein, *LDL* low- density lipoprotein, *SHR* subdistribution hazard ratio

## Discussion

This prospective study showed that abdominal artery calcification was common in PD patients. Older age, longer PD duration, diabetes, and previous CVD were correlated with AACS in prevalent PD patients. Furthermore, high AACS was an independent predictor of subsequent cardio-cerebral vascular disease and all-cause mortality in the study cohort.

Abdominal artery calcification is particularly common in dialysis patients, with the overall prevalence ranging from one third to more than 80% [4, 14–16]. We reported an abdominal artery calcification prevalence of 57.2% in our PD patients, in correspondence with published meta results in Asia population [4]. There were a few cross-sectional studies suggesting that abdominal artery calcification might be less common in PD patients compared to HD patients [14], while other studies reported no advantage of one modality over the other [4, 17].

Vascular calcification is a sophisticated process and our results suggest both demographics and comorbidity might contribute to its development. Age is a traditional risk factor for vascular calcification [16, 18, 19]. With body aging, pathologies promoting calcium deposits such as lipid deposition and decrement of smooth muscle and elastin might occur in vessel walls [18, 20]. Prolonged exposure to uremic toxins and biocompatible dialysate could lead to sustained activation of calcification inducers along with the down-regulation of calcification inhibitors [21, 22]. It has been shown that the prevalence of vascular calcification in incident PD patients increases from 47 to 56% after 1 year on dialysis [23]. Diabetes closely associated with vascular calcification, and insulin resistance, oxidative stress, and hyperglycemia are possible mechanisms participating in [24, 25]. In consist with several other studies, a previous history of CVD was found to be associated with higher AACS [16, 19]. No relationship was found between phosphate, PTH and calcium and abdominal artery calcification in the present study, which were reported as accelerators of calcification in some observational studies [26, 27]. The lack of association may relate to the time-dependent high variability of these biochemical makers since our analysis only included the parameters measured at the enrollment.

Imaging examinations to investigate vascular calcification include CT scan, abdominal plain X-ray, mammography and vascular ultrasound [5–7]. The optimum diagnostic technique for vascular calcification remains unsettled. CT based imaging is outstanding in accuracy and reproducibility, yet plain radiography is a convenient and inexpensive alternative, and it delivers substantially lower radiation [28]. The predictive value of AACS based on lateral lumbar X-ray film for CVD and mortality has been validated in the general population [29, 30]. Moreover, it has been shown that AACS correlates with coronary artery calcification score by electron beam CT in HD population [31].

Previous studies had provided evidence of abdominal artery calcification as a prognostic factor in ESRD patients. The Calcification Outcome in Renal Disease (CORD) Study, a large prospective study conducted in 47 European dialysis centers, explored the relationship between AACS and all-cause mortality as well as nonfatal cardiovascular events in a cohort of 1084 dialysis patients, of which the vast majority were on HD. The investigators found AACS to be an independent predictor of the adverse outcomes [9]. A few studies focused on PD populations and found that abdominal artery calcification is a risk factor of both mortality and CVD occurrence, but were carried in a rather small population or with restricted age-bracket [32, 33]. This relatively larger cohort study with longer follow-up periods might help for the extrapolation of these findings in PD population. Recently, Mäkelä S et al. reported that severe arterial calcification (AACS≥7) rather than moderate calcification (1 ≤ AACS< 7) was associated with adverse outcomes in Finnish PD patients [34]. Comparatively, out study found that even mild calcification (AACS≤4) can double the risk of MACCE and death compared to the ones without abdominal artery calcification, and regular screening of abdominal artery calcification to identify PD patients with excess CVD risks are therefore suggested.

Therapeutic interventions to prevent the progression of vascular calcification may be of great value in these patients identified with high AACS, which requires the long-term implementation of systematic interventions targeting at multiple pathogenic components [8, 35]. Control of mineral metabolism was considered critical to reducing the vascular calcification progression, and non-calcium-based phosphate binders and/or 1.25 mmol/L calcium dialysate are suggested. Compared with 1.75 mmol/L calcium dialysate, utilization of 1.25 mmol/L calcium dialysate tended to decrease serum calcium level without inducing high turn-over bone lesion in PD patients [36, 37]. A meta-analysis reported that in CKD stage 3-5D patients, Agatston scores were significantly lower in patients treated with non-calcium-based phosphate binders than those on calcium-based binders, with a mean score difference of − 95.26 (95%CI − 146.68 to − 43.84) [38]. Excessive use of activated vitamin D should be avoided in patients with severe vascular calcification, and parathyroidectomy is recommended for severe secondary hyperparathyroidism [35]. Limited evidence implied that no beneficial effect of cinacalcet treatment on arterial stiffness despite the remarkable lowering of the PTH level in PD patients [39, 40], and clearly further randomized controlled trials with better design are needed.

CVD is highly prevalent in ESRD patients and is the leading cause of death in dialysis patients that accounts for more than 50% of all-cause mortality [9]. The presence and extent of arterial calcification are regarded as one of the major determinants for CVD morbidity and mortality through multifaceted pathogenesis. In CKD population, arterial calcification is characterized by lesions that occur in the medial vascular wall, which exacerbates arterial stiffness termed as arteriosclerosis [41]. Arterial rigidity is measurable through pulse wave velocity (PWV). Numerous studies in CKD and ESRD patients have reported this positive correlation between increased arterial calcification and faster PWV [42]. It is responsible for escalated systolic blood pressure, decreased diastolic blood pressure, and widened pulsatile pressure [43]. Increased wave reflections and high pulse pressure are independent risk factors for mortality [44]. Elevated systolic pressure aggravates ventricle afterload and oxygen consumption, leads to left ventricular hypertrophy, and results in myocardial fibrosis, arrhythmias and congestive heart failure^,^ [45]. 1 g/m^2.7^/month increase in left ventricular mass index was associated with a 62% increase in the incident risk of cardiovascular events in HD patients [46]. Decreased diastolic pressure limits coronary perfusion and promotes myocardial ischemia, which in turn urges myocardial infarction and lethal arrhythmias [47].

Coronary artery calcification (CAC) is the traditional marker for coronary atherosclerotic burden and direct causal factor in the pathogenesis of ischemic heart diseases [48]. There is an observed association between aortic stiffness and CAC [49]. The stiff arterial wall may be subjected to greater shear and intraluminal stresses as the result of increased pulsatile pressure, inducing vessel wall damage and endothelial cell dysfunction which are the crucial steps of coronary atherosclerosis [50].

There are several limitations in our study. First, the single-center study design limits the generalizability of our results. Second, as mentioned above we only included laboratory parameters and AACS at the baseline of the study. The lack of serial assessments leads to inevitable confounders. The presence of the longitudinal changes in the time-dependent variables may provide more solid information.

## Conclusions

In summary, the present study implies the important role of AACS measured by lateral lumbar X-ray to independently predict all-cause mortality and MACCEs in a large Chinese PD cohort. This cheap and easy method facilitates the evaluation of vascular calcification in patients undergoing PD and the stratification of the risk of cardiovascular and cerebrovascular disease.

## Data Availability

The datasets analyzed during the current study are available from the corresponding author on reasonable request.

## Abbreviations

CVD: Cardiovascular disease
AACS: Abdominal aortic calcification score
MACCE: Major adverse cardiac and cerebrovascular events
PD: Peritoneal dialysis
HD: Hemodialysis
CKD: Chronic kidney disease
ESRD: End-stage renal disease
hs-CRP: high-sensitivity C reaction protein
iPTH: intact parathyroid hormone
AKP: Alkaline phosphatase
Kt/V: Total weekly urea clearance
CT: Computed tomography
ACS: Acute coronary syndrome
PCI: Percutaneous coronary intervention
CABG: Coronary artery bypass grafting
SD: Standard deviation
IQR: Interquartile range
PWV: Pulse wave velocity
